# The Benefits and Challenges of Conducting Primate Research in Different Settings

**DOI:** 10.3390/ani13010133

**Published:** 2022-12-29

**Authors:** Stacy M. Lopresti-Goodman, Brandon Villatoro-Sorto

**Affiliations:** Department of Psychology, School of Social and Behavioral Sciences, Marymount University, 2807 North Glebe Road, Arlington, VA 22207, USA

**Keywords:** primates, research ethics, welfare, cognition, behavior, laboratories, zoos, sanctuaries, field studies

## Abstract

**Simple Summary:**

Primates have been used in research for the past hundred years as a window into our shared evolutionary history, to learn more about their unique behavioral and psychological processes, and as models for human behavior and diseases. Today, primate research takes place in laboratories, zoos, sanctuaries, and the wild. Research settings that most closely resemble primates’ natural habitats provide the best insights into their lives, produce more valid results, and reduce potential negative impacts on their well-being. This paper provides a novel overview of current non-invasive psychological research, and explores the benefits and challenges of conducting research with primates in different settings. It also suggests ways to help improve primate research to mitigate some scientific and ethical concerns.

**Abstract:**

Internationally, primate research takes place in laboratories, zoos, sanctuaries, and the wild. All of these settings present unique advantages and challenges in terms of methodology, translatability, animal welfare, and ethics. In this novel commentary, we explore the scientific and ethical benefits and drawbacks of conducting non-invasive psychological research with primates in each setting. We also suggest ways to overcome some of the barriers. We argue that while there may be greater experimental control in laboratory-based research, settings that more closely mirror primates’ natural habitats are generally better suited to meet their specialized needs. More naturalistic research settings, including field studies, may also circumvent some ethical concerns associated with research in captivity, and yield more ecologically valid data.

## 1. Introduction

Nonhuman primates (hereafter referred to as primates) share a close phylogenetic relationship with humans, resulting in developmental, behavioral, and psychological similarities. Primates have been used in research for the past hundred years to learn more about their unique behavioral and psychological processes, and as models for human behavior and diseases because of our shared evolutionary history [1,2,3,4,5,6,7,8,9,10].

Today, primate research takes place in various settings that each present unique advantages and limitations in terms of methodology, translatability, animal welfare, and ethics [1,4,10,11,12,13,14]. Research environments that most closely resemble primates’ wild living conditions are considered to be best suited for evaluating their natural psychological processes [1,2,15,16,17].

In this article, we review the benefits and challenges of conducting non-invasive psychological research in laboratories, zoological parks (hereafter referred to as zoos), sanctuaries, and the wild. We argue that while there may be greater experimental control in laboratory-based research, non-laboratory settings are better suited to meet the specialized needs of primates, may yield more ecologically valid data, and are less ethically problematic.

## 2. Laboratory-Based Research

Historically, laboratories have been preferred by many primate researchers because they offer the benefit of a high degree of experimental control. The primates most often used in laboratory research are macaques (*Macaca*) [7,18,19,20,21]; however, baboons (*Papio*), chimpanzees (*Pan troglodytes*), squirrel monkeys (*Saimiri*), vervet monkeys (*Chlorocebus*), and marmosets (*Callithrix*) are also used [6].

In 2019, the most recent year data were available, there were 68,000 primates used in research in the U.S. [22]. There were approximately 8000 primates used in research facilities in the European Union (E.U.) [19]. In China, 28,000 primates were reportedly used in laboratory research [23]. Due to current reporting systems, it is impossible to determine exactly how many primates were used in non-invasive versus invasive research.

While some primates in laboratories are wild-caught, most are commercially-bred offspring of wild-caught monkeys or live in breeding colonies at research facilities [6,9,24,25]. Recently, restrictions on the breeding and use of primates in laboratories have been proposed and implemented in various countries [26,27,28]. In laboratories, primates may live in outdoor corrals consisting of over 100 animals of mixed age and sex groups, in smaller social groups with access to indoor–outdoor enclosures, or may be singly housed in indoor cages if required for the experimental protocol [7,29,30].

Most of the current non-invasive psychological research in laboratories focuses on cognition, social behavior, and improving the welfare of their captive-living primates [1,4,7,24]. A recent systematic review analyzed over 1000 articles on primate cognition published in the past 15 years [4]. It found that 44% of them were authored by individuals at research centers and universities [4]. Contemporary cognitive research focuses on attention, metacognition, communication, numerical cognition, perception, illusions, episodic and prospective memory, theory of mind, and self-control [2,31,32,33]. Researchers are also interested in comparative cognition, or how evolutionary pressures have resulted in similarities and differences across primate species [11,33,34]. Others have focused on the establishment of social bonds, prosociality, inequity aversion, fairness, empathy, cooperation, and competition [1,7,33].

Although the topics listed above are no doubt important areas of research, several characteristics of primate research in laboratories may confound the data [1,2,15,17]. These factors include routine housing, husbandry, and experimental conditions.

With regard to routine housing, the strictly controlled laboratory environments do not mirror the complexity of the physical or social environments that primates have evolved in [2,4,9,16,17,32]. This includes constrained social relationships, a lack of sufficient environmental or cognitive enrichment, and many of the natural stressors they would encounter in the wild, such as selecting a mate or evading predators [9,17]. The ecological pressures that shape the physiological and psychological development of primates in laboratories are markedly different from the pressures faced in the wild, and therefore may result in different behavioral outcomes [2,17].

In an attempt to improve laboratory conditions, researchers may use structural, passive, or active sensory enrichment [35]. Some researchers argue that experimentation itself can act as a form of enrichment for laboratory-living primates [36]. Even “enriched” environments, however, are often insufficiently complex. This can result in primates’ inability to engage in species-typical behaviors, and may lead to boredom and the performance of stereotypical and abnormal behaviors [2,7,17,36]. Abnormal behaviors are behaviors not typically observed in the wild, or occur with greater frequency and intensity in captivity [36,37]. Researchers have argued that the presence of abnormal behaviors may not be indicative of current compromised welfare. They have suggested that instead they may be socially learned [36]. Conversely, the absence of abnormal behaviors does not imply good welfare [36]. If primates are to be kept in captivity, then it is important to conduct research on which behaviors are good indicators of negative welfare, and how to minimize and treat these behaviors [36]. The mere presence of these abnormal behaviors, however, may confound results and influence validity, reliability, and replicability [2,17,38].

One way to potentially reduce the effects abnormal behaviors have on research findings is to use “Natural Laboratory Complexes”. This is where primates can live in large social groups and move freely between indoor and outdoor enclosures. These more natural laboratory environments may allow for observing richer, more naturalistic behaviors while maintaining some level of experimental control [16], and reduce the expression of abnormal behaviors [36].

Even the “best” laboratory environments are unable to replicate the rich environmental and social lives of primates in the wild. For example, Šlipogor et al. [39] investigated personality structures in wild and captive populations of marmosets. They found that the wild monkeys’ personality structure included additional dimensions beyond those in captivity. They suggest that different living conditions yield different social settings that can affect the behavioral outcomes being assessed [32]. This raises scientific concerns about making “population-to-species generalizations” when using any captive populations [2].

In addition to impoverished environments affecting outcomes, another challenge of conducting laboratory research is that primates in laboratories often participate in a number of different studies throughout their lifetime [9]. Repeated use can result in confounding variables influencing their performance on different cognitive or behavioral tasks [11,14]. Humans and habituation to experimental stimuli can also greatly influence experimental outcomes [2,17,32]. For example, captive orangutans (*Pongo*) living in social groups interacted with experimental objects differently, and had different outcomes on cognitive tasks, than their solitary wild counterparts [32]. Others have criticized laboratory-based cognition experiments for investigating what an animal *can do*, but not what they have evolved to do, or may typically do in the wild [2,34].

Laboratory-based psychological studies are not only problematic from a research validity standpoint, but also from an ethical standpoint. For example, most primates are euthanized when no longer needed, though there are efforts underway to retire animals to sanctuaries whenever possible [40].

Laboratory primate use also has implications for the stability of wild populations [41]. In 2022, the International Union for Conservation of Nature (I.U.C.N.) listed the long-tailed macaque (*Macaca fascicularis*) as “endangered” for the first time, in part because of their exploitation for research purposes [42]. Additionally, the growing costs of keeping primates in laboratories make alternative research settings increasingly desirable [16].

For all of the previously described reasons, non-laboratory-based primate research is gaining traction [43]. The more naturalistic environments and larger social groups in zoos may avert some of the welfare and validity concerns of laboratories [44]. The increased attention research in zoos has been garnering makes this topic particularly timely [10,23].

## 3. Research in Zoos

Zoos, by definition, are places where animals live in captivity and are put on display for public exhibition [45,46]. Estimates state that over 700 million people visit zoos each year [47], with the majority stating their primary purpose is to have an outing or be entertained [47,48,49]. In the past few decades, zoos have tried to shift their image away from places of entertainment to centers for conservation, education, and research [47,49,50,51,52,53,54].

There are approximately 5500 primates living in North American zoos accredited by the Association of Zoos and Aquariums (A.Z.A.), including lemurs, New and Old-World monkeys, and great apes [23]. While the exact number of primates in E.U. zoos is unknown, according to a 2021 European Association of Zoos and Aquaria (E.A.Z.A.) report, there are more than 1700 great apes and 2000 squirrel monkeys, in addition to a variety of other primates, in E.A.Z.A. facilities [55]. Primates are the most-studied taxa in zoos [10,56], with apes comprising approximately two-thirds of all of the primate subjects studied [23].

One benefit of zoo-based research is that primates in zoos often live in species-typical social groupings and semi-naturalistic environments designed to mimic their wild habitats. This includes access to the outdoors, climbing structures, and natural substrate [23,57]. This maintains some degree of ecological validity for research on captive primates [56]. Given that it is still a highly controlled captive environment, it may allow for a higher degree of experimental control than sanctuaries or the wild [57]. Another benefit of conducting research at zoos is that they provide “taxonomic diversity” which is beneficial for comparative purposes [23,44,52,57,58].

Historically, a challenge of conducting zoo-based primate research was that zoos were primarily interested in studies on welfare, husbandry, nutrition, and veterinary practices [10,59]. Large-scale research on psychological processes seemed unfeasible given a lack of dedicated, knowledgeable zoo staff, as well as monetary constraints [56]. Researchers also assumed certain logistical and statistical limitations would arise with testing primate cognition in social settings, such as reduced sample sizes and difficulty identifying individual responses [52,60]. These ideas have been increasingly challenged with zoos operating as viable research settings for basic and applied research with primates. Researchers have demonstrated they are capable of maintaining relatively high experimental control in zoos [4,23,44,52,57].

This increase in psychological research at zoos is due in part to increases in research-center–zoo partnerships. Some notable examples include the University of St. Andrews and Royal Zoological Society of Scotland, the Max Planck Institute for Evolutionary Anthropology and Leipzig Zoo in Germany, and Georgia State University, Emory University, and Zoo Atlanta in the U.S. [4]. Additionally, some zoos in the U.S. have created their own research centers, including the Lester E. Fisher Center for the Study and Conservation of Apes at the Lincoln Park Zoo in Chicago, IL, USA, or the “Think Tank” at the Smithsonian National Zoological Park in Washington, DC, USA. [4]. Indeed, a recent analysis found that 25% of publications on primate cognition in the past 15 years have come from zoos [4]. This research includes investigations of short-term memory [11], theory of mind, cognitive bias [61], categorization, and metacognition [57]. Others have investigated the social transmission of behaviors [13], personality [56,62,63,64,65], cooperation [2], and within-group dynamics through social network analyses [59,66].

Although “physical” tasks continue to be used to test cognition in zoos [4], technological advances have changed the way primate research is conducted via the use of computer touchscreens, eye-tracking devices, and automated behavioral monitoring systems [23,57,59,67]. One benefit of using technology such as touch-screens in zoos is that it may allow for the primates to choose when to participate in studies. This is not necessarily the case in laboratories, where they are often kept captive for the sole purpose of conducting research. Despite this form of data collection facilitating research with acquiescent primates, some argue it creates concerns about the scientific validity of such studies, as mentioned above [44]. Are researchers assessing primates’ abilities, or merely assessing potential capabilities [57]?

One challenge of conducting research in zoos is that small sample sizes may make the generalizability of results difficult [56]. One way to counteract this is to collect data from multiple social groups at different zoos, using standardized ethograms or electronic data-collection applications [44,56,59]. For example, ZooMonitor is an application designed by the Lincoln Park Zoo to record animal behavioral data. This allows for more reliable data collection across sites, and is available to zoos at little to no cost [68]. WelfareTrak^®^, a welfare-monitoring tool that allows caregivers to complete online weekly surveys about animal welfare [69], may help increase the standardization of research questionnaires when assessing the behavior and personality of zoo-living primates [56]. Replicating studies across zoo sites is also a good way to validate experimental findings.

Another challenge of conducting research in zoos is the potential impact that experimental methods may have on the primates’ welfare. For example, some authors argue that conducting research in zoos can act as a form of enrichment and increase primates’ welfare, particularly when they voluntarily participate in studies [23,57,67,70]. Other authors report mixed results, ranging from positive to negative [4,47]. This may include frustration caused by unsuccessful completion of a computerized task which can result in negative affect expressed as self-directed behaviors [71,72].

Another concern is the fluctuating crowd sizes and varying noise levels related to the constant presence of visitors. Some authors argue visitors have a positive impact, acting as a form of enrichment. Other researchers argue that visitors have a neutral or negative influence [56,67,73,74,75,76,77,78,79,80,81,82,83,84,85,86,87,88]. For example, researchers have found that high percentages of primates in zoos, including chimpanzees, gorillas (*Gorilla*), gibbons (*Hylobates syndactylus*), orangutans, and a number of monkey species, are negatively affected by visitors. This includes having increased cortisol levels, aggression, fear, and engaging in more stereotypical and abnormal behaviors. They may also exhibit a decrease in species-specific behaviors [56,73,74,85,86,87,89,90,91].

A recent study comparing abnormal behaviors in chimpanzees in laboratories, zoos, and sanctuaries found chimpanzees in zoos were actually more likely to engage in abnormal behaviors than those in other settings [75]. The authors found that the individuals came from a variety of different sources (e.g., zoo-born, wild-caught, laboratories), had different early rearing experiences, and lived in various social and environmental conditions prior to relocation to the zoo. This makes causation of their abnormal behaviors difficult to attribute. Regardless of the cause, the relatively high presence of abnormal behaviors in zoo-living chimpanzees is ethically problematic, may compromise the interpretation of the data and results, and is therefore a challenge of conducting research in that setting [2].

Another welfare concern is that zoos often restrict primates’ movement so they can be easily viewed in enclosures that are much smaller than the space used by their wild counterparts [75,81]. Researchers have argued that it is not the size of the space available, but the complexity that impacts primate welfare [81,92]. Studies have found that the introduction of visual barriers, providing the primates opportunities for control over their environment, and allowing them to retreat from visitors, can reduce negative effects associated with visitor presence, including decreased aggression and abnormal behaviors [86]. Some zoos have also implemented positive reinforcement training and environmental enrichment to enhance the performance of naturalistic behaviors and decrease the performance of abnormal or stereotypic behaviors [51].

As explained above, there are many benefits and challenges of conducting research in zoos. One way that zoos and laboratories are similar is that they both highly manage the lives of their primates. This includes when they can access different aspects of their enclosures, the size of the space, and their diet [81]. Primates in the wild spend a large percentage of their time foraging for food. In captivity, they are regularly provided with food, some of which may be unnatural to their diet, including monkey chow [81]. Using unpredictable feeding schedules, and scattering food around their enclosures, however, can help promote foraging behavior [81]. Zoos also house multiple species of animals within a close proximity, including predators and prey, which can result in increased stress [81]. This is often not the case in laboratories or sanctuaries, where single species are housed.

While zoos have made great strides in increasing their capacity for research, and researchers have developed ways to avoid some of the potential scientific challenges of working in that setting, there are still many researchers and members of the public who have ethical and welfare concerns about zoos [47,49,53]. Primate sanctuaries are an alternative environment that has seen an increase in psychological research in the past decade.

## 4. Research in Sanctuaries

While laboratories and zoos breed or acquire primates for the purpose of using them for research or to keep on display, sanctuaries play a critically important role in housing primates who are no longer wanted or needed from other settings. Sanctuaries in range countries provide life-long care for wild primates who have been orphaned, abandoned, or who would be unable to survive on their own in the wild [10,93]. In the U.S. and E.U., sanctuaries provide homes for primates who were former pets, used in entertainment, or released from research [10,94,95,96,97,98,99,100]. Many sanctuaries only house one species of primates, reducing some of the stressors primates in zoos may experience being housed next to predators [10].

Sanctuaries also differ from laboratories and zoos in that they do not buy, sell, trade, or breed primates [94,98]. They are environments that prioritize the well-being of their residents above other potential interests, including research. Therefore, most sanctuaries and accrediting bodies have strict standards for what kinds of research activities can be conducted [94,98]. They often prohibit anything which could compromise the individuals’ well-being or would interfere with their daily activities [10]. Other sanctuaries might not allow any research to be conducted given early potential trauma their primates may have experienced [4]. Sanctuaries that do allow research may restrict activities to surveys or purely observational studies on applied behavior, cognition, culture, and welfare [10,100,101]. In the past few years, however, there has been increased interest by sanctuaries in allowing a wider variety of research activities [102].

There is a rich array of research occurring at primate sanctuaries [10], which demonstrates that researchers have been able to overcome some of the challenges associated with restrictive research requirements. Recent studies in sanctuaries include research on social networks, dominance hierarchies, social learning [103], communication, play behavior, cooperation, helping behavior, altruism, perspective taking [10], social dynamics [96], and social interactions [4]. Others have investigated friendship and trust [104], personality [65,95,96,105], welfare [10], psychological health, and mood and anxiety disorders [93,100,101]. A recent analysis found that 6% of studies on primate cognition published in the past 15 years have been conducted in sanctuaries [4]. This includes research on problem solving, tool use, reasoning, theory of mind, [106], short-term memory [11], long-term memory [107], attention [108], spatial memory, thinking, learning, perception, and emotion [10]. With regards to experimental apparatuses, cognitive researchers in sanctuaries tend to use tools, puzzle boxes, and manipulatable objects, more so than touch screens [4,10,105]. The wide array of topics listed above demonstrates that sanctuaries have found ways to allow robust research to occur with their primates without compromising their mission.

Some benefits of working with sanctuaries include that they tend to have much larger populations of primate species than laboratories or zoos, with more naturalistic social and physical environments [4,10,102,109,110]. They also greatly restrict public access, reducing any negative impacts large crowds of visitors may have on the primates. This makes it possible to study more naturalistic behaviors than other captive settings, and may provide greater ecological validity [4,10].

There are additional logistical challenges for researchers in sanctuaries that may not be encountered in laboratories or zoos. For example, for the benefit of animal welfare, sanctuary enclosures are not designed for easy public viewing; therefore, primates may occupy parts of their enclosure where they cannot be seen. Using technology such as ZooMonitor or cameras placed around the enclosures can help ease data collection [10,110]. An additional challenge is that sanctuaries typically do not have the infrastructure or staff capable of conducting research, because of a lack of time, formal research training, or money, given most are funded by private donations [10,110]. One way to circumvent some of these challenges is to form research partnerships, such as that between the Lincoln Park Zoo and ChimpHaven in Louisiana, or Fundació Mona and the University of Girona in Spain, to facilitate rigorous research programs, using both onsite observations and analysis of data from cameras off site [10,99,102,110,111]. These partnerships provide opportunities to study primates in semi-naturalistic environments, and any fees the sanctuaries may charge the researchers can go to caring for primates.

Another potential challenge of conducting research at sanctuaries is that atypical early histories may serve as a confounding factor in any psychological studies. These factors may include being captured in the wild, atypical rearing conditions, a lack of interactions with conspecifics, or repeated use in biomedical research before being relocated to a sanctuary [75]. These primates may have experienced psychological or physical trauma [112]. The increased stress from these adverse experiences can also result in changes in brain morphology [113] and the performance of abnormal behaviors, such as hair-plucking, coprophagy, regurgitation, and reingestion [1,36,75,100]. This is similar to a limitation of conducting research with zoo-living primates. Engaging in abnormal behaviors in sanctuaries may not be indicative of current compromised welfare, but may carry over from previous experiences [75]. Importantly, research has shown that increased time in sanctuaries results in the decreased performance of abnormal or aberrant behaviors, decreased vigilance, and increased relaxation and socialization [100,114]. One suggestion for working with primates in sanctuaries may be to allow for an acclimation period to their new life prior to conducting studies with them.

Regardless of the setting, we must acknowledge that laboratories, zoos, and sanctuaries are all forms of captivity that are highly managed, but with varying degrees of restrictions in terms of social groups, enclosure size, and complexity. As a result, these sites may still negatively affect primates’ welfare, including restricting autonomy [112], freedom of choice [115], the diversity of dynamic social groups [32], and the natural stressors encountered in the wild [17]. Additionally, primates in all of these settings are forced to interact with humans. This can lead to “enculturation effects” resulting in changes in their behavior and cognitive performance on experimental tasks [5,32].

As researchers, we must ask ourselves: is conducting research for the sake of our curiosity worth any potential harm to the populations we are studying, even if the harms are unintended or non-obvious [2]? If we acknowledge that no form of captivity can replicate the richness of their natural habitats and lives, but still have important psychological, behavioral, or welfare-based research we wish to pursue, then working in primate sanctuaries may be the most ethical option. If we are interested in understanding primates’ natural psychological processes and behaviors without the confounding factors that come from captivity, then conducting field research may be the best way to mitigate these potential problems.

## 5. Research in the Wild

In order to best understand the complex abilities and lives of primates, scientists may want to study these processes in wild populations that developed under the ecological pressures and rich social dynamics they have evolved in [5,17]. Wild primate populations are much larger than those in captivity, consisting of multi-male, multi-female social groups, representing all ages and developmental stages [5,113]. Their more diverse social situations and habitats allow researchers to study a wider array of behaviors in populations that have not been used in repeated experiments [113]. It also provides researchers with more information about the evolutionary origins of their abilities required for survival [4,5,11,32].

Studying primates in situ is also particularly important given that as of 2015, approximately 497 primate species and 698 taxa are considered threatened [116], most wild primate species are under-documented, and they face the rapid decline of their habitat [117]. Conducting more research with them in the wild can provide us with greater information about their behavior, habitat needs, and life history which can help improve conservation efforts. It can also help us enrich the habitats of those living in captivity [16]. Further, field studies are necessary to provide external validity for some research performed in captivity, including in comparative cognition [113].

Recent field studies have focused on communication, social knowledge, social cognition [5], social networks, self-awareness, deception, [118], dominance [1,4,15,118], and personality [119]. Field researchers have also studied coordination, cooperation, and play behaviors [13], in addition to more traditional cognition, such as learning [120] and tool development and use [118].

Cognitive research in the field tends to be more observational in nature, focusing on what animals know and its adaptive value, rather than what they might be capable of doing [4,15]. However, there has been increased interest in the use of experimentation in the wild. This allows for experimental manipulation while retaining ecological validity [5,120]. This research includes studying problem solving with puzzle boxes, social learning [5,15], associative learning [120], communication, and cooperation [5]. This research may even be accomplished through computerized touch systems where individuals living in social groups can voluntarily visit throughout their day, in combination with live-stream videos [16]. It is important that experimental introductions do not alter the culture of the group being studied. Most researchers who work with primates in the wild would agree that interactions, interventions, and disturbances by humans should be minimized to ensure the primates’ welfare and follow codes of best practice established by primatological societies [16,121].

One challenge of field research is that there may be small sample sizes of participants, given neophobia for experimental objects or hesitancy to approach humans [120]. If the primates are not motivated to participate by food rewards, they cannot be coerced to do so, unlike in captivity [113]. Additionally, there is reduced experimental control, which can restrict the kind of research being conducted.

A benefit of conducting research with primates in their natural habitats is that it can lead to new directions for researchers to investigate [57]. Moreover, as others have argued, while experiments have traditionally been thought of as the “gold standard”, researchers can never control all possible confounding variables [17,113]. Consequently, we should think of observational research and experimental studies as sitting on a continuum [113]. Indeed, Janmaat [113] provides a framework for studying cognition in the field using chimpanzees as her target primate species.

There are still ethical concerns regarding field research. Primarily, they relate to interactions between humans and primates, increasing the potential for disease transmission and habituation [12,113,120,121]. The provisioning of wild populations is controversial [5], and can result in increased human and nonhuman primate conflict [122]. Human presence may also inadvertently impact primates’ ranging patterns and intra- and intergroup dynamics [121]. When conducting field studies, researchers should avoid surprising their subjects and alert them to their presence with nonthreatening species-specific behaviors before and during any habituation process [121].

One way to reduce interactions with humans is using electronics, remote sensors, camera traps, and audio triangulation to study primates in the wild [16,113,123]. While the financial cost of traveling to field sites or setting up technology to study their behavior remotely may appear prohibitive, it might actually cost less than using primates in laboratories. Research facilities incur significant costs from breeding, transportation, housing, husbandry, and staff salaries [16]. If data are collected in the wild and shared electronically, it can also increase collaborations between individuals at different institutions working on a variety of primate research projects, and the cost of experimental apparatuses may be distributed [16]. Despite the logistical challenges and potential lack of experimental control, field research seems the best option to conduct ethical, ecologically valid, non-invasive psychological research on primates who are socially situated [4,16,57].

## 6. Conclusions

As discussed above, primate research takes place in various settings around the world, including in laboratories, zoos, sanctuaries, and the wild. Each of these settings has different advantages and disadvantages for researchers, including ease or difficulty of data collection, level of experimental control, generalizability, and validity of results. Equally important, each setting offers different levels of social and environmental complexity, and interference by and interaction with humans, which has implications for primates’ welfare. To our knowledge, this is the first article comparing the benefits and challenges of primate research in all possible settings. It is our opinion that research settings that most closely mirror primates’ natural habitats are generally better suited to meet their specialized needs, may circumvent some ethical concerns associated with research in captivity, and yield more ecologically valid data.

Much of the research cited here has revealed important insights into primates’ psychological needs and abilities. Understanding this should therefore prompt increased scrutiny of how research with them in captivity or the wild might impact their well-being [2,9,16,25]. For instance, given ethical and scientific concerns, stricter criteria have been imposed for housing and using chimpanzees in non-invasive and invasive research [124,125], while greater restrictions on primate use generally have been proposed by others [9,25,126]. In the U.S. and E.U. the concept of the “3 Rs” (Replacement, Reduction, and Refinement) is an important principle that guides research with animals in laboratories [2,127]. The “3 Rs” should be applied to primate research in all settings [128]. Reducing the numbers of primates could be accomplished with increased collaborations with researchers across sites. Primate research could be refined using technology for monitoring behavior. More recently, a fourth R, Retirement, has been introduced [40,129]. Retiring, instead of euthanizing, healthy primates no longer needed in laboratories is the ethical thing to do, and could result in increased subjects for continued non-invasive psychological research in sanctuaries.

Increased public awareness about the welfare of animals in zoos might also one day lead to a future where fewer primates are kept in those captive conditions, similar to concerns that have prompted reevaluation of breeding and using orcas in sea parks [49,130].

Given the dwindling wild populations of primates, and the rapid decline of their habitats, research in sanctuaries and field studies may become the only viable option for certain research in coming decades. Therefore, it is important that we continue to develop novel ways to conduct non-invasive psychological research on primates in non-laboratory settings that provide the highest-quality data while helping protect and promote their wellbeing. Future investigations could examine trends in where primate research is conducted and how specific kinds of primate studies could be improved by moving to a non-laboratory setting.

## Data Availability

Not applicable.
